# An innovative OSCE clinical log station: a quantitative study of its influence on Log use by medical students

**DOI:** 10.1186/1472-6920-12-111

**Published:** 2012-11-12

**Authors:** Judith N Hudson, Helen Rienits, Linda Corrin, Martin Olmos

**Affiliations:** 1 Deputy Head of School, School of Rural Medicine, University of New England, Armidale, NSW, 2351, Australia; 2 Graduate School of Medicine, University of Wollongong, Wollongong, NSW, 2522, Australia; 3 Higher Education, University of Melbourne, Melbourne, Victoria, Australia

**Keywords:** Medical education, Electronic reflective clinical log, Assessment, OSCE log station

## Abstract

**Background:**

A Clinical Log was introduced as part of a medical student learning portfolio, aiming to develop a habit of critical reflection while learning was taking place, and provide feedback to students and the institution on learning progress. It was designed as a longitudinal self-directed structured record of student learning events, with reflection on these for personal and professional development, and actions planned or taken for learning.

As incentive was needed to encourage student engagement, an innovative Clinical Log station was introduced in the OSCE, an assessment format with established acceptance at the School. This study questions: How does an OSCE Clinical Log station influence Log use by students?

**Methods:**

The Log station was introduced into the formative, and subsequent summative, OSCEs with careful attention to student and assessor training, marking rubrics and the standard setting procedure. The scoring process sought evidence of educational use of the log, and an ability to present and reflect on key learning issues in a concise and coherent manner.

**Results:**

Analysis of the first cohort’s Log use over the four-year course (quantified as number of patient visits entered by all students) revealed limited initial use. Usage was stimulated after introduction of the Log station early in third year, with some improvement during the subsequent year-long integrated community-based clerkship. Student reflection, quantified by the mean number of characters in the ‘reflection’ fields per entry, peaked just prior to the final OSCE (mid-Year 4). Following this, very few students continued to enter and reflect on clinical experience using the Log.

**Conclusion:**

While the current study suggested that we can’t assume students will self-reflect unless such an activity is included in an assessment, ongoing work has focused on building learner and faculty confidence in the value of self-reflection as part of being a competent physician.

## Background

There is a considerable literature in undergraduate, postgraduate and continuing health professional education on reflective logs or portfolios suggesting that they are an ideal tool to capture students’ learning experiences and/or to foster learning in a work or professional study environment [1-6]. Outcomes reported include improvements in knowledge and understanding, increased self-awareness and engagement in reflection and improved student–tutor relationships, assistance in knowledge management processes and connection between learning at organisational and individual levels, and career-long management of continuing professional development activity.

However, there is wide variation in portfolio content, use and assessment and much debate about what constitutes a portfolio [7,8]. While it has been defined as a collection of evidence gathered and maintained for a specific purpose [9], several authors claim that a key issue to differentiate a portfolio from a dossier of evidence is that the former incorporates reflection on learning [10-12]. The stance that capacity for reflection is a key component of competency in professional practice is supported by the educational theory, the ‘reflective practitioner’ [13]. Schön’s epistemology of professional practice was not only based on reflection-on-action or thinking *after* solving problems, but also on reflection-in-action or thinking *while* problem solving [14]. He advised that an important part of the competence and artistry in practice of skilful practitioners was their ability to think about what they are doing while they are doing it, including reflection on situations of uncertainty, instability, uniqueness and conflict [14]. Reflective learning in medical education has since been shown to improve professionalism and clinical reasoning, with reflective practice contributing to continuous practice improvement and better management of complex health systems and patients [15].

The widespread uptake of reflective portfolios has been attributed to the emphasis on reflective practice and the trend towards competency-based education [3]. The growing use of portfolios in postgraduate and continuing education, and reports that a reflective portfolio increased undergraduate students’ self-confidence in their ability to complete a portfolio in the future [16,17], suggests that we should introduce students to learning portfolios during undergraduate training. However uptake has been tempered by the perception that portfolios are resource intensive for both learners and those who rate them. For example, the time commitment of keeping a portfolio deterred medical students from engaging with the process unless required to do so by the demands of assessment [18,19]. Also faculty need to make decisions about the quantity and quality of the entries and how best to reliably assess these [20-24].

### Context for the research

In February 2007 the first cohort of students commenced their studies at the Graduate School of Medicine in Wollongong, where the problem-based medical curriculum is organised around 93 core clinical presentations. Early and longitudinal clinical experience is another feature of the 4-phase outcomes-base course. Phase 1 includes fortnightly local hospital and community placements, while Phase 2 comprises hospital-based rotations in medicine, surgery, paediatrics, maternal and women’s health and mental health. Phase 3 is a year-long integrated clerkship based in general practice, where community clinical experience is integrated with hospital emergency and ward-based patient care. Phase 4 consists of a national or international elective, a selective and a student-internship. Progression at the end of each phase depends on success in a range of assessments, blue-printed to the course learning outcomes, in the competency-based assessment programme. The latter includes an Objective Structured Clinical Examinations (OSCEs) at the end of Phases 2 and 3.

In this new graduate-entry medical school, with a mission to develop competent clinicians and address the maldistribution of medical workforce in Australia [25], a Clinical Log was introduced as part of a student learning portfolio, to monitor the quantity and quality of student early and longitudinal clinical experience provided in regional and rural communities [26]. It was designed as a longitudinal self-directed structured record of student learning events, with reflection on these for personal and professional development, and actions planned or taken for learning. The Log aimed to facilitate development of students’ reflective skills with recording of increasing level of involvement and confidence over time. For each record, fields of entry comprised the time and location of the patient ‘visit’; de-identified data on the patient presentation; key discerning features; the diagnosis and differential; any procedures performed; reflection on learning needs and strategies for addressing them; and student and supervisor comments. The Log complemented other curriculum and assessment activities on critical self-reflection, the latter including written personal and professional development reflections completed throughout the course, and students were encouraged to elaborate on recorded Log experiences for these formal tasks. Faculty also wished to use the Log to monitor student achievement of curriculum learning outcomes and identify areas that may need to be more thoroughly addressed, and to provide evidence of curriculum coverage and engagement for internal and external accreditation requirements. To achieve both these purposes, student engagement with the Log was crucial.

While some students embraced electronic recording and reflection on their early clinical experiences, initial uptake was low. Advice that a comprehensive reflective record of undergraduate clinical experience may provide a competitive edge when applying for postgraduate training positions, was not sufficient motivation at this stage. Barriers to Log use included the time commitment required to complete the Log; it was not well integrated with the curriculum; and Log aims, objectives and requirements were not well understood by all students and Faculty. The large range of supervising clinicians/mentors in dispersed learning sites made this more difficult to achieve. Moreover, while several senior clinicians embraced completion of a dossier of evidence, they were less comfortable with the reflective component, and did not encourage use by students. While steps were initiated to address these challenges and modifications were made to simplify the process of Log entry, further incentive was needed. To encourage student usage and a habit of reflection on recorded experiences, an innovative Clinical Log station was introduced in the OSCE. Adding a station to the OSCE, an assessment format with established acceptance at the School, was seen as a cost-effective way to achieve this aim. This study, aiming to review the impact of the Clinical Log OSCE station, addressed the following research question:

How does an OSCE Clinical Log station influence Log use by students?

## Methods

### Introduction of OSCE station

Given the dispersed nature of the clinical placements, the Clinical Log played a critical role in correlating students’ clinical experience with the curriculum. To encourage student use a decision was made to introduce a Clinical Log station in the Phase 2 OSCE. A Formative OSCE had been scheduled in March 2009, about three months before the first Summative Phase 2 OSCE, so students and assessors could be ‘trained’ on the nature of this examination: its content, format and standard-setting procedure, and to give student feedback on their progress to date. This was the ideal occasion to ‘trial’ a Clinical Log OSCE station.

**Figure 1:**
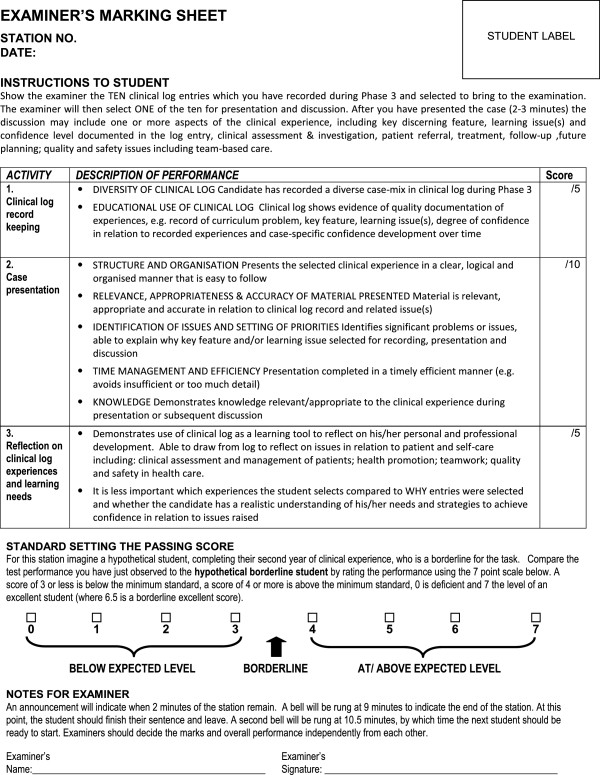
Clinical Log OSCE station marking sheet.

The Clinical Log station, developed by the Director of Clinical Education (JNH) in a similar format to other OSCE stations, comprised a marking sheet and station instructions and aims. It aimed to foster longitudinal recording and reflection on clinical experience and identification of significant learning issues in relation to all aspects of patient and self-care, health promotion, teamwork and quality and safety. The scoring process sought evidence of educational use of the log, and an ability to present and reflect on key learning issues in a concise and coherent manner, as illustrated in Figure 1.

Assessors scored performance using the following three main criteria: quantity and diversity of recorded experiences; presentation of case; and reflection on development issues in relation to the presentation. A systematic approach for generating the scores was implemented. The pass-fail cut score for the Log station was calculated using the borderline regression method of standard setting [27], as for the other 12 stations. Students and assessors were briefed on the station and standard setting procedure, and also had online access to the station information and aims, prior to the examinations. The Clinical Log station was then included in the subsequent Summative Phase 2 and Phase 3 OSCEs.

### Post OSCE feedback

While all students were offered one-on-one feedback on OSCE station performance, those who failed the station received an email encouraging them to come for feedback, and all students in this category (and the few who were absent for the formative OSCE), attended for follow-up. Each section of the marking sheet was discussed with these students, for example how each was valued according to the marks, response to written assessors comments and identification of strategies to respond to these.

The study received ethics approval from the University Human Ethics Research Committee.

## Results

Seventy students completed the Formative OSCE (95% of the Phase 2 cohort), with all students completing the Phase 2 (N = 74) and Phase 3 Summative OSCEs (N = 68) respectively. Student absence for the initial Phase 2 Formative OSCE and attrition prior to the Phase 3 OSCE in June 2009 explains the varying student numbers at each of these examinations.

### Performance on the OSCE Log station

Cohort performance on this station improved with each OSCE experience, as evidenced by the increasing station cut point and mean of the cohort performance (Table 1).

**Table 1:** Comparison of average station scores and cut points for the Clinical Log station in the Formative and Summative OSCEs (2009, 2010)

	**Number of students (N)**	**Station cut point (pass mark)**	**Failed the Clin. Log Station**	**Failed the OSCE**	**Mean score for student cohort (Log station)**	**Standard deviation (Log station)**
		**(Maximum = 20 marks)**			**(Maximum =20 marks)**	
**Phase 2 Formative OSCE (April 2009)**	**70**	**8.89**	**4**	**1**	**13.00**	**2.77**
**Phase 2 Summative OSCE (June 2009)**	**74**	**10.03**	**3**	**3**	**14.32**	**2.21**
**Phase 3 Summative OSCE (July 2010)**	**68**	**10.97**	**0**	**1**	**16.06**	**1.65**

### Log usage

**Figure 2:**
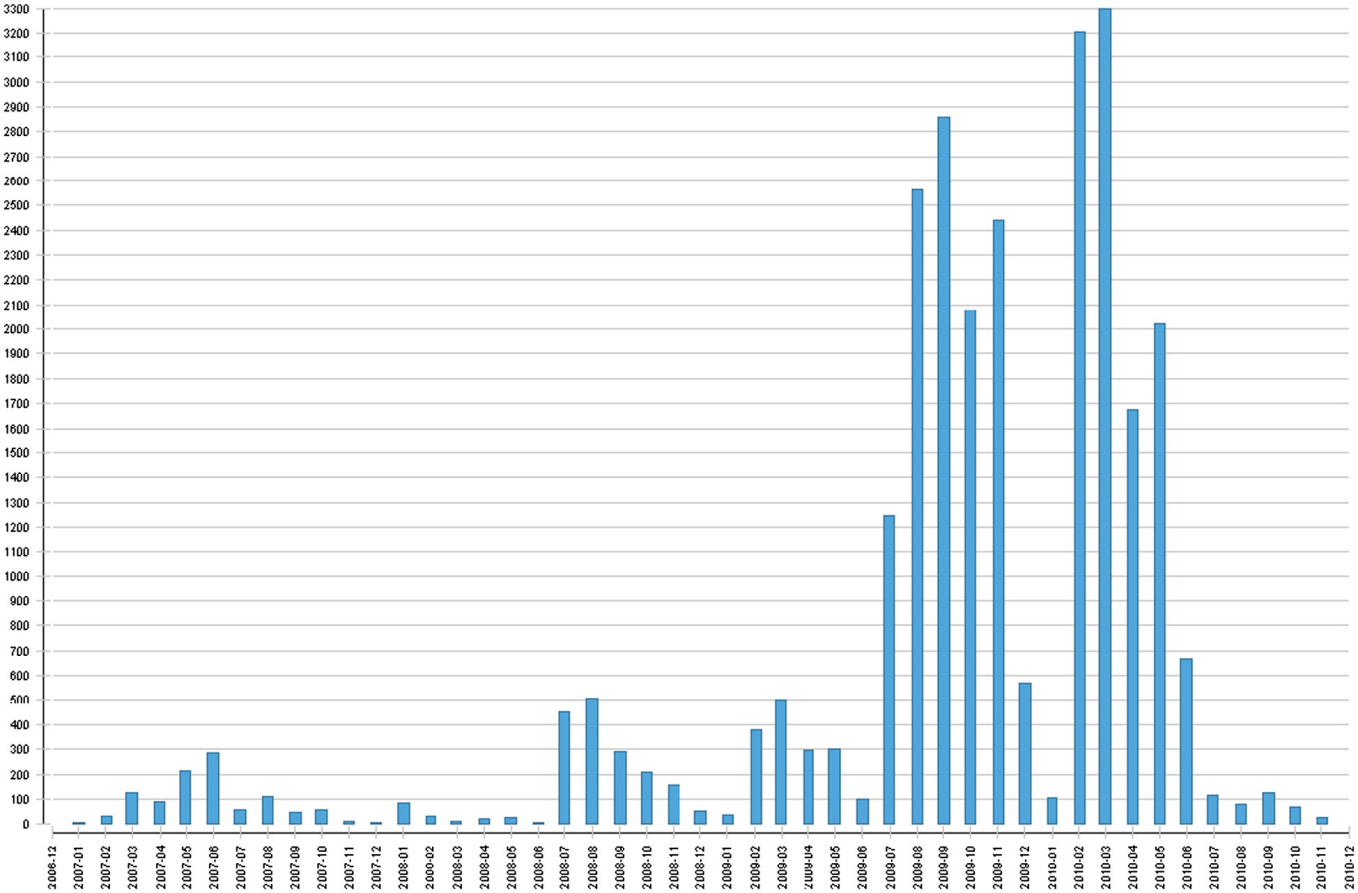
Cohort Clinical Log entries 2007–2010 (year-month vs number patient visits, all students).

Examination of cohort Log use over the four years of the course (quantified as the number of patient visits entered by all students) revealed there was limited use for the first two years, with a small flurry of activity when the students commenced their Phase 2 hospital-based speciality rotations in July, 2008. Log use was stimulated following introduction of the OSCE station early in the third year, and following the June holiday break, students engaged with the log to a greater extent during Phase 3. However, after the Phase 3 OSCE in June 2010, very few students continued to enter and reflect on clinical experience using the Clinical Log (Figure 2).

Continuous monitoring of log usage revealed there was a rush of log entries in the period prior to the Formative Phase 2 OSCE in late March, 2009; the Summative Phase 2 OSCE in mid-June 2009, and the Summative Phase 3 OSCE in early July, 2010. While many entries were recorded at the time of the patient visit, close analysis revealed that there was a flurry of data entry immediately preceding each OSCE, and that many of these entries were delayed relative to the patient visit.

### Quantity of reflection recorded in the log

**Figure 3:**
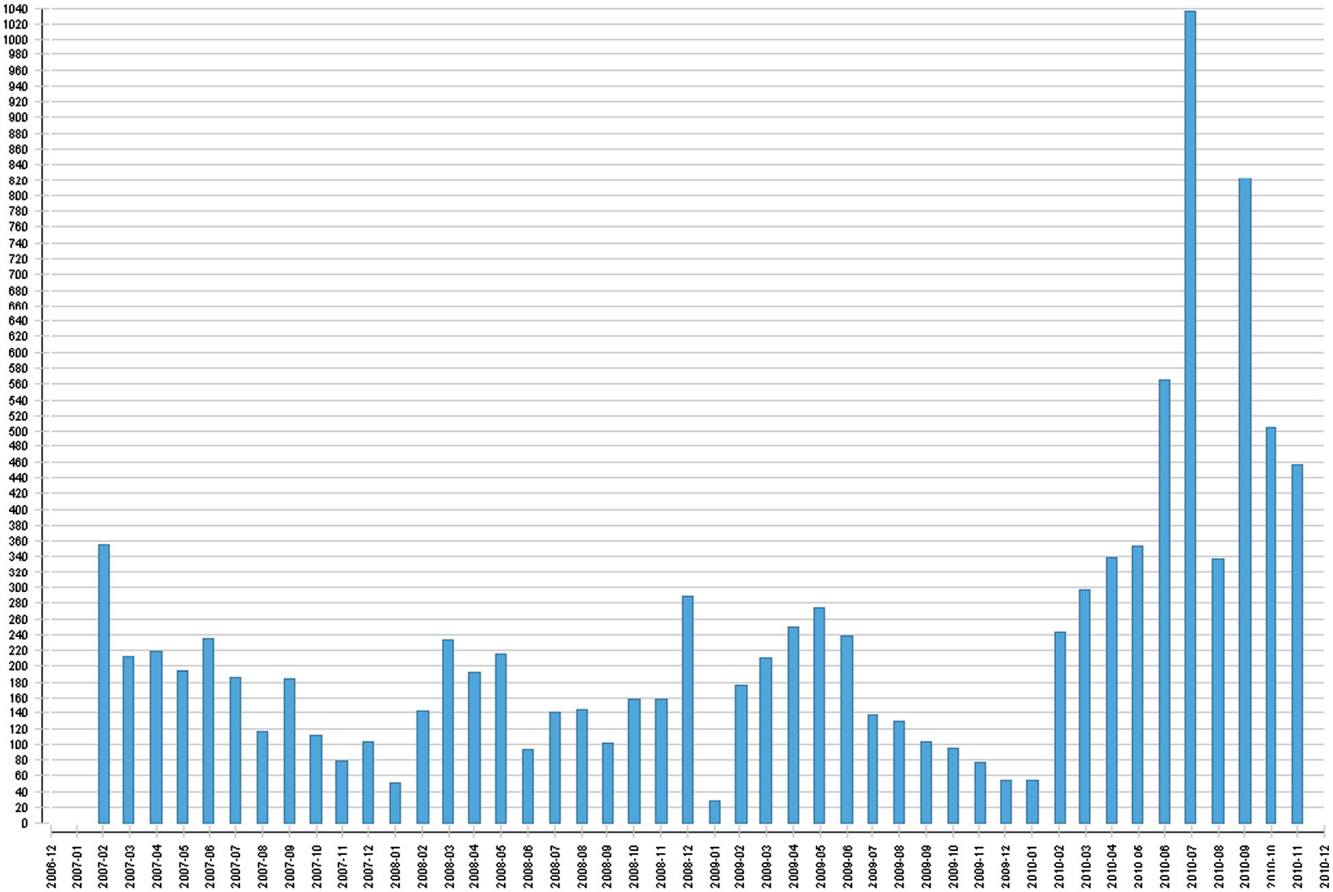
Cohort quantity of reflection over course 2007–2010 (year-month vs mean number of characters in all three ‘reflection fields’ per Log entry).

**Figure 4:**
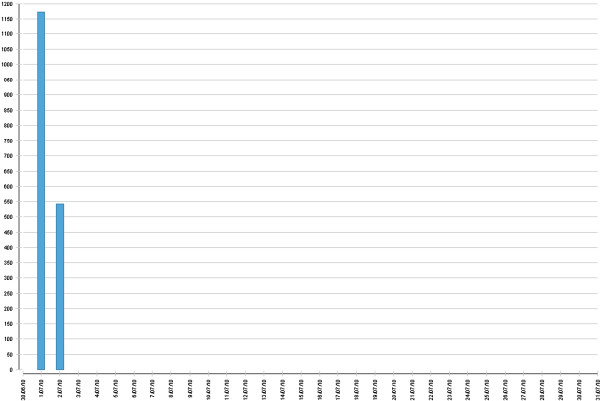
Cohort quantity of reflection, prior to final OSCE on July 3rd 2010 (month-day vs mean number of characters in all three ‘reflection’ fields per Log entry).

Student reflection, quantified by the mean number of characters in the ‘reflection’ fields per entry, peaked just prior to the Phase 3 OSCE (Figure 3). Closer analysis of the month of July entries reveals a large increase in data-entry in the reflection fields on July 1st and 2nd, in the two days preceding the Phase 3 OSCE (Figure 4).

Following the final OSCE on July 3rd, log use including reflection was limited to a very small number of students.

## Discussion

At the start of a new graduate-entry medical school in Australia, an electronic Clinical Log was implemented, as part of a learning portfolio, to capture students’ learning experiences and foster learning in a range of clinical settings throughout the course. It also aimed to develop a habit of critical reflection while learning was *taking place*, and provide feedback to students and the institution on learning progress. As such, the Clinical Log had high face validity with utility for formative assessment but sound psychometrics were needed for high stakes summative purposes [20]. To address reliability issues such as high variability of scoring between examiners, the Log assessment was carefully introduced with clear articulation of criteria to both students and assessors. Experienced trained assessors, who understood the purpose of the assessment and expected student performance, were used. This study, the first in a series reporting on outcomes and challenges associated with the Clinical Log, showed that most students had a low initial level of engagement with the Log until motivated by inclusion of a Clinical Log station in the end-of-Phase OSCEs. It demonstrated the well-known fact that ‘assessment drives learning’. However the initiative encouraged student engagement with the school’s aim to foster student reflection for professional reasons.

The improvement in cohort performance on the Log station with subsequent OSCEs was attributed to the following: assessment criteria were communicated to students and assessors prior to each exam; a formative assessment experience was offered prior to the summative testing; and post-exam feedback and remediation were offered to all students, especially borderline and failing students.

While it seemed that the Log OSCE assessment was the major motivator for recording of clinical experiences, it was pleasing to observe greater use by Phase 3 students throughout the year-long community-based integrated placement. This may have been due to greater exposure to ‘undifferentiated patients’ and a growing appreciation of the value of self-reflection.

For this paper only the quantity of student reflection in the Log has been analysed. It seemed that for most students inclusion of the reflective criteria in the OSCE station was necessary for student engagement in this desired professional activity. The quality of the reflections in the Log needs further evaluation. The fact that many students were only motivated to record and reflect clinical encounters when the OSCE assessment approached suggested that most reflection occurred *on* rather than *in*, professional action [14]. Reflection *on* action can occur when the student enters and reflects on each patient-interaction in the Log, or shares Log experiences with preceptors, or peers and tutors. Delayed Log entry may have limited impact on reflection *on* action but it is likely to significantly impede reflection *in* action. The longitudinal placement is valued as it offers students the benefits of long-term patient follow-up (continuity of care experiences), and ongoing reflection on diagnosis and management decisions as the presentation unfolds (reflection *in* action). While the Log allowed recording of continuity of care for individual patients, use of this facility and reflection *in* action requires Log entry closely related to the patient-interaction, rather than delayed entry as assessment approaches. The planned development of a mobile Clinical Log application may facilitate student engagement with more reflection *in*, as well as *on* action.

The School continues to monitor student and clinician feedback on the Clinical Log, addressing issues that have discouraged use. More guidance is being offered on expectations and the educational use of the Log, showing it as an activity for, rather than being in competition with, learning. With the growing use of e-portfolios and logs in undergraduate and postgraduate education and recognition of the value of self-reflection for professional competency [15], the clinicians who supervise and/or mentor students have been offered more training on providing constructive feedback on learners’ personal and professional development, and reflection on this. This was deemed important for those who embraced completion of a dossier of evidence but were less comfortable with the reflective component in the Log and assessment. Potentially the investment in faculty professional development will have benefits for learners in the vertical continuum of medical education, and faculty themselves (as most teachers contribute to both undergraduate and postgraduate medical education, and may use logs/portfolios themselves in continuing medical education).

Further work is underway to review the quality, as well as the quantity of the reflections and correlate these with learner academic success. This should help strengthen the evidence base for use of electronic reflective logs as part of learning portfolios in undergraduate medical education and build learner confidence in the value of reflection for developing professional artistry. It will be interesting to further investigate at what stage students appreciate the value of self-reflection as part of being a competent physician.

Recent work on different aspects of a portfolio approach to competency-based assessment has reported the value of giving students the responsibility of ‘interpreting, selecting and combining formative assessments received during the year, to document their performance in a learning portfolio for summative decisions’ [28-30]. The authors advise that this has helped students to internalise the self-regulation process, potentially more valuable for professional development than an extrinsic driver of portfolio use, such as assessment.

## Conclusions

While the current study suggested that we can’t assume students will self-reflect unless such an activity is included in an assessment, subsequent efforts to embed the reflective log in the students’ learning environment should facilitate ongoing student engagement. Ongoing work has also focused on building learner and faculty confidence in the value of self-reflection as part of being a competent physician.

## Competing interests

The authors declare that they have no competing interests.

## Authors’ contributions

JNH, HR, LC and MO have all made substantial contributions to conception and design of the study. LC and MO acquired the data and completed data analysis, and all authors were involved in data interpretation. JNH drafted the manuscript. All authors have revised it critically for important intellectual content and have given final approval of the version to be published.

## Pre-publication history

The pre-publication history for this paper can be accessed here:

http://www.biomedcentral.com/1472-6920/12/111/prepub
